# Sociodemographic factors associated with anticipatory perceived labor vulnerability related to the Russia–Ukraine war among workers in Latin America

**DOI:** 10.1186/s13031-026-00823-z

**Published:** 2026-08-02

**Authors:** Christian R. Mejia, Victor Serna-Alarcón, Jamil Cedillo-Balcázar, Scherlli E. Chamorro-Espinoza, Jaime A. Yáñez, Neal M. Davies, Leonel Vega-Perez, Camilo Vega-Useche

**Affiliations:** 1 Universidad de Huánuco., Huánuco, Perú; 2 Universidad Privada Antenor Orrego, Piura, Trujillo, Peru; 3 Hospital de la Amistad Perú Corea Santa Rosa II-2, Piura, Peru; 4 Dirección Provincial de Salud de Loja, Ministerio de Salud Pública, Loja, Ecuador; 5 Asociación Médica de Investigación y Servicios en Salud, Lima, Peru; 6 Escuela de Posgrado, Universidad Internacional Iberoamericana, Campeche, Mexico; 7 University of Alberta, Edmonton, Canada; 8 Universidad Pedagógica y Tecnológica de Colombia, Tunja, Colombia; 9 Fundación Santa Fe de Bogotá, Bogotá, Colombia

**Keywords:** Occupational health, War, Workers, Latin America, Risk perception, Job insecurity, Perceived vulnerability, Armed conflict

## Abstract

**Introduction:**

The COVID-19 pandemic disrupted labor markets in Latin America, a region characterized by high informality, socioeconomic inequality, and uneven social protection. Shortly afterward, the Russia–Ukraine war generated global uncertainty regarding inflation, commodity prices, supply chains, and economic stability. However, the extent to which geographically distant armed conflicts are perceived as potential labor-related threats by workers in structurally vulnerable regions remains insufficiently understood.

**Objective:**

To identify sociodemographic factors associated with self-reported high anticipatory perceived labor vulnerability related to the Russia–Ukraine war among workers in Latin America.

**Methodology:**

A cross-sectional, analytical, multicenter secondary analysis was conducted among workers in Latin American countries during the first weeks of the Russia–Ukraine conflict. Participants were asked about their perceptions of the potential impact of that conflict on their work, reflecting an anticipatory and perception-based outcome rather than direct war exposure or objectively measured labor-market change. High anticipatory perceived labor vulnerability was defined as reporting “quite a lot” or “a lot” on a five-category perception item. Descriptive and analytical statistics were performed. Results: Among 1,118 workers, 25.2% perceived quite a lot of labor-related impact from the Russia–Ukraine war, and 34.6% perceived a lot of impact, corresponding to 59.8% of the analytical sample being classified as having high anticipatory perceived labor vulnerability. Regarding work expectations, 33.3% anticipated a decrease in work, 27.5% expected work to remain the same, 25.9% reported that they could lose their job, and 13.3% anticipated an increase in work. A total of 62.0% anticipated that salaries would decrease for most workers. In the main adjusted model, male sex was associated with a lower prevalence of high anticipatory perceived labor vulnerability (aPR: 0.77; 95% CI: 0.69–0.85; p < 0.001). Age and country of residence were not independently associated with the outcome.

**Conclusion:**

A high proportion of surveyed workers reported anticipatory perceived labor vulnerability related to the Russia–Ukraine war. The most consistent sociodemographic association was observed for sex. These findings should not be interpreted as evidence of objective labor-market effects, but as an exploratory assessment of subjective labor-related risk appraisal during an early period of geopolitical uncertainty.

**Supplementary Information:**

The online version contains supplementary material available at https://doi.org/10.1186/s13031-026-00823-z.

## Introduction

The COVID-19 pandemic produced major disruptions in labor markets, work organization, employment, working hours, household income, and perceived job security, particularly in Latin America, where high informality and socioeconomic inequality increased workers’ vulnerability to economic shocks [1–7]. These disruptions occurred in a context of structural labor vulnerability, uneven access to social protection, and heterogeneous recovery after the acute phases of the pandemic. As a result, workers in the region may have been especially sensitive to subsequent global crises capable of affecting employment expectations, purchasing power, and economic stability.

The Russian invasion of Ukraine in February 2022 generated a major international crisis with legal, political, humanitarian, economic, trade, food security, and energy-related consequences [8–11]. Although Latin American workers were not directly exposed to the armed conflict, the war occurred in an interconnected global economy and generated uncertainty regarding inflation, commodity prices, supply chains, and macroeconomic stability. From the perspective of conflict and health, such crises may produce indirect transnational spillover effects that extend beyond direct physical exposure to violence. These spillovers may include perceived threats to economic stability, employment expectations, household security, and psychosocial well-being in geographically distant populations.

A more explicit conceptual framework is needed to understand these subjective responses. Risk perception is shaped not only by objective exposure, but also by uncertainty, perceived controllability, prior vulnerability, social amplification, and the salience of crisis-related information. Similarly, economic insecurity and perceived job insecurity are recognized psychosocial stressors in occupational and social epidemiology. Therefore, in populations not directly exposed to armed conflict, perceived labor vulnerability may reflect a subjective appraisal of potential economic and occupational consequences rather than actual labor-market deterioration. This distinction is particularly important during the early phase of a geopolitical crisis, when media attention, public uncertainty, and information exposure may amplify perceived threat.

In this context, anticipatory perceived labor vulnerability should be understood as self-reported appraisals of possible downstream occupational consequences rather than as objectively measured labor outcomes or direct war-related harm. This clarification is essential because perception-based outcomes may capture risk appraisal, uncertainty, and perceived vulnerability in response to global economic shocks, but they cannot establish whether actual changes in employment, income, or working conditions occurred. Therefore, this study aimed to identify sociodemographic factors associated with self-reported high anticipatory perceived labor vulnerability related to the Russia–Ukraine war among workers in Latin America.

## Methodology

### Study design and population

Workers from Latin America participated in a survey conducted via Google Forms during the first weeks after the Russian invasion of Ukraine in February 2022. This survey was part of a larger study that initially assessed mental health in a broad Latin American population [12], from which this secondary data analysis was derived. The present analysis was restricted to participants with work-related information and focused on perceived possible labor vulnerability related to the conflict. The study employed a cross-sectional, analytical, multicenter design.

Adults who lived and worked in the surveyed countries during the first weeks of the conflict and who consented to participate were eligible for inclusion. For this secondary analysis, “workers” were operationally defined as participants who had complete information for the core work-related variables in the parent dataset, including years of work experience, type of work, job role, and responses to the labor-impact perception items. The analytical sample included 1,118 participants with complete data for the primary outcome, sex, age, country of residence, type of work, job role, work experience, and the work and salary perception variables. Six participants had missing information for occupation/labor category and were retained in analyses that did not require this variable.

A non-random snowball sampling strategy was used. Initial participants were recruited through contacts in different companies and labor sectors and were then asked to share the survey with other eligible workers. Because recruitment occurred through open snowball dissemination, the number of individuals who received the survey link, the number who actively declined participation, and the reasons for non-participation were not available in the secondary dataset; therefore, a conventional response rate could not be calculated. Consequently, the sample should not be interpreted as representative of workers in Latin America or of workers within any participating country. The recruitment strategy may have preferentially reached individuals with internet access, greater digital connectivity, higher educational attainment, younger age, urban residence, or greater engagement with social and professional networks. These characteristics may have influenced both participation and perceived labor-related vulnerability during the early phase of the conflict.

The survey platform did not provide sufficient information in the secondary dataset to reconstruct all duplicate-prevention procedures; however, the analytical database was audited and contained no duplicate participant identifiers or exact duplicate records. Accordingly, all prevalence estimates reported in this manuscript should be interpreted as sample-specific descriptive estimates rather than population-level estimates for Latin American workers.

To assess the adequacy of the sample size, statistical power was calculated using Stata software. Power was greater than 80% for most analyses, except for certain subgroups, including the labor category (56%), mixed work subtype (18%), type of work (76%), history of depression (8%), and suicidal ideation (18%). These results should therefore be interpreted with caution and considered exploratory.

### Study variables

The primary outcome was participants’ perceived overall impact of the Russia–Ukraine conflict on their work. For reporting purposes, the main perception item can be translated as: “To what extent do you think the Russia–Ukraine war could affect your work?” This outcome was assessed using a single exploratory survey item with five ordinal response options: none, little, fair, quite a lot, and a lot. The item was designed for the parent survey and was not part of a previously validated scale of job insecurity, economic insecurity, occupational risk perception, or psychosocial vulnerability. No formal validation study of this item was available in the present secondary analysis. Therefore, the outcome should be interpreted as an exploratory measure of anticipatory perceived labor vulnerability rather than as a validated measure of job insecurity or objective labor-market impact.

For the main analysis, responses were dichotomized into “quite a lot” or “a lot” (category of interest) versus “none,” “little,” or “fair” (comparison category) in order to identify workers reporting a comparatively high level of anticipatory perceived labor vulnerability. This analytical decision facilitated estimation of prevalence ratios, although it may have reduced information contained in the original ordinal scale and may have obscured gradients of perceived vulnerability across response categories. For this reason, an ordinal logistic regression using the original five-category response was conducted as a sensitivity analysis and interpreted as exploratory because the proportional odds assumption was not formally tested. Two additional perception variables were evaluated descriptively: expected changes in work (increase, remain the same, decrease, or job loss) and expected changes in salary (increase for most, increase for a few, remain the same, decrease for a few, or decrease for most).

Additional covariates included sex (male or female), age (years), labor category (health vs. other), work experience (years), type of work (face-to-face, virtual, or mixed), job role (operator/field vs. administrative), and country of residence. The countries with sufficient frequency for individual analysis were Colombia, Paraguay, Peru, Bolivia, Ecuador, and Mexico. Responses from additional Latin American countries with low frequencies—including Panama, Brazil, Argentina, Cuba, Guatemala, Chile, and Honduras—were grouped into the category “Other” for analytical purposes, as their sample sizes were insufficient for separate analysis. A sensitivity analysis using the original 13-country variable was also performed to evaluate the influence of country grouping. History of anxiety, depression, stress, and suicidal ideation were derived from single-item binary self-reported variables (yes/no) from the parent survey dataset and were treated as covariates in this secondary analysis. The exact wording and recall period of these mental health items were not available in the secondary analytical dataset; therefore, they should be interpreted as self-reported history rather than clinically confirmed diagnoses or validated measures of current mental health status.

### Procedures

The study protocol was submitted to and approved by the Ethics Committee of Antenor Orrego Private University (official letter No. 0418–2022). Original data were filtered to include only worker responses, and quality control was performed to ensure completeness and accuracy. The database was then cleaned, categorized, and organized in Microsoft Excel 2019 before being exported to Stata version 18 for statistical analysis.

### Data analysis

Descriptive statistics were generated for the socio-occupational characteristics of the participants, including frequencies and percentages for categorical variables and measures of central tendency and dispersion for quantitative variables (age and work experience). Labor-related perceptions were summarized according to the three work-related perception questions. Given the non-probabilistic sampling strategy, all descriptive estimates were interpreted as characteristics of the analytical sample rather than as population-level estimates for Latin American workers.

Bivariate analyses were conducted using the dichotomized perception of work impact as the dependent variable and each covariate separately. Crude prevalence ratios (cPRs), 95% confidence intervals (95% CIs), and p-values were estimated using generalized linear models of the Poisson family with log link and robust variance. The multivariable model was specified a priori based on conceptual relevance and potential confounding, including sex, age, and country of residence. This model was selected as the primary model because these variables are temporally antecedent sociodemographic characteristics available for the entire analytical sample, whereas occupational and mental health variables could represent downstream correlates of perceived vulnerability, related constructs, or alternative explanatory pathways. Stress was therefore not retained in the primary model because it may represent a related psychosocial construct or mediator rather than a clear confounder of the association between sociodemographic characteristics and perceived labor vulnerability. Adjusted prevalence ratios (aPRs), 95% CIs, and p-values were estimated using the same modeling approach. Because of the cross-sectional design and the subjective nature of the outcome, these models were interpreted as estimating associations with high anticipatory perceived labor vulnerability, not causal effects.

To address residual confounding and reviewer concerns, several sensitivity analyses were performed. First, a model additionally adjusted for self-reported stress was fitted. Second, an occupationally adjusted model included labor category, work experience, type of work, and job role. Third, a full exploratory model additionally included self-reported anxiety, depression, stress, and suicidal ideation. Fourth, an ordinal logistic regression model was fitted using the original five-category outcome. Because the proportional odds assumption was not formally tested, this ordinal model was interpreted as exploratory and was not used for primary inference. Multicollinearity was assessed using variance inflation factors from an equivalent linear regression specification. An exploratory model using robust standard errors clustered by country was also examined; however, because only seven country groups were available, clustered estimates were interpreted cautiously and were not used as the main inferential approach. Formal interaction analyses were not conducted because several subgroup strata were small, the study was not designed to test effect modification, and interaction estimates would likely have been unstable. Potential heterogeneity by sex, country, occupation, or work modality should therefore be evaluated in future studies with larger and more representative samples. Additional details and results of the sensitivity analyses are presented in the Supplementary Methods and Supplementary Tables 1 and 2.

All analyses were performed in Stata 18. The primary outcome and main model covariates had complete data for 1,118 participants. Occupation/labor category had six missing values; therefore, models including this variable used 1,112 participants.

## Results

As shown in Tables 1 and 55.5% (620) of participants in the analytical sample were female, the median age was 25 years (IQR: 22–30), and the median work experience was 4 years (IQR: 2–8). Most participants worked in non-health occupations (78.1% among those with available occupation data), had face-to-face work (76.7%), and held administrative roles (52.5%). In addition, 26.0% reported anxiety, 18.6% reported depression, 24.5% reported stress, and 3.4% reported suicidal ideation.

**Table 1:** Socio-occupational characteristics of the analytical sample surveyed in the early phase of the Russia–Ukraine war

Variable	*n* (%) or summary
**Sex**	
Female	620 (55.5)
Male	498 (44.5)
**Age**,** years**	
Mean ± SD	28.1 ± 9.5
Median (IQR)	25 (22–30)
**Labor category**	
Non-health occupation	868 (78.1)*
Health occupation	244 (21.9)*
Missing	6 (0.5)
**Work experience**,** years**	
Mean ± SD	6.8 ± 8.2
Median (IQR)	4 (2–8)
**Type of work**	
Face-to-face	857 (76.7)
Virtual	76 (6.8)
Mixed	185 (16.5)
**Job role**	
Operator or field	531 (47.5)
Administrative	587 (52.5)
**Country**	
Other	153 (13.7)
Colombia	225 (20.1)
Paraguay	178 (15.9)
Peru	216 (19.3)
Bolivia	152 (13.6)
Ecuador	106 (9.5)
Mexico	88 (7.9)
**Self-reported anxiety**	
No	827 (74.0)
Yes	291 (26.0)
**Self-reported depression**	
No	910 (81.4)
Yes	208 (18.6)
**Self-reported stress**	
No	844 (75.5)
Yes	274 (24.5)
**Self-reported suicidal ideation**	
No	1080 (96.6)
Yes	38 (3.4)

Values are presented as n (%) unless otherwise indicated. SD: standard deviation; IQR: interquartile range. *Percentages for labor category were calculated using the available denominator, *n* = 1,112; six participants had missing information for occupation/labor category

According to Table 2 5.2% of participants perceived quite a lot of overall labor-related impact from the Russia–Ukraine war, and 34.6% perceived a lot of impact, resulting in 59.8% of participants being classified as having high anticipatory perceived labor vulnerability. Regarding work expectations, 33.3% anticipated a decrease in work, 27.5% expected work to remain the same, 25.9% reported that they could lose their job, and 13.3% anticipated an increase in work. Regarding salary expectations, 62.0% perceived that salaries would decrease for most workers. These estimates describe the non-probabilistic analytical sample and should not be interpreted as population prevalence estimates for Latin American workers.

**Table 2:** Labor-related perceptions in the analytical sample during the early phase of the Russia–Ukraine war

Variable	*n* (%)
**Overall perceived labor impact**	
None	102 (9.1)
Little	154 (13.8)
Fair	193 (17.3)
Quite a lot	282 (25.2)
A lot	387 (34.6)
**Expected change in work**	
Would lose job	290 (25.9)
Would remain the same	307 (27.5)
Would decrease	372 (33.3)
Would increase	149 (13.3)
**Expected change in salary**	
Would remain the same	171 (15.3)
Would decrease for most	693 (62.0)
Would decrease for a few	186 (16.6)
Would increase for most	17 (1.5)
Would increase for a few	51 (4.6)

Percentages were calculated using the full analytical sample, *n* = 1,118. High anticipatory perceived labor vulnerability was defined as reporting either “quite a lot” or “a lot” in the overall perceived labor impact item. Because the sample was non-probabilistic, percentages should be interpreted as sample-specific descriptive estimates rather than population-level estimates

According to Table 3, high anticipatory perceived labor vulnerability was associated in crude analyses with sex (*p* < 0.001), job role (*p* = 0.001), Ecuador compared with the grouped “Other” country category (*p* = 0.007), self-reported anxiety (*p* = 0.014), self-reported depression (*p* = 0.009), and self-reported stress (*p* = 0.001). Age (*p* = 0.064), work experience (*p* = 0.076), and suicidal ideation (*p* = 0.094) did not reach conventional statistical significance in the crude analysis.

**Table 3:** High anticipatory perceived labor vulnerability related to the Russia–Ukraine war according to socio-occupational factors in the analytical sample

Variable	No high vulnerability	High vulnerability	cPR	95% CI	*p*-value
**Sex**					
Female	202 (32.6)	418 (67.4)	Ref.		
Male	247 (49.6)	251 (50.4)	0.75	0.67–0.83	< 0.001
**Age**,** years**	25 (22–32)	25 (22–30)	0.99	0.99–1.00	0.064
**Labor category**					
Non-health occupation	349 (40.2)	519 (59.8)	Ref.		
Health occupation	97 (39.8)	147 (60.2)	1.01	0.90–1.13	0.898
**Work experience**,** years**	4 (2–10)	3.5 (1.5–8)	0.99	0.99–1.00	0.076
**Type of work**					
Face-to-face	339 (39.6)	518 (60.4)	Ref.		
Virtual	37 (48.7)	39 (51.3)	0.85	0.68–1.06	0.155
Mixed	73 (39.5)	112 (60.5)	1.00	0.88–1.14	0.980
**Job role**					
Operator or field	185 (34.8)	346 (65.2)	Ref.		
Administrative	264 (45.0)	323 (55.0)	0.84	0.77–0.93	0.001
**Country**					
Other	60 (39.2)	93 (60.8)	Ref.		
Colombia	86 (38.2)	139 (61.8)	1.02	0.86–1.20	0.846
Paraguay	80 (44.9)	98 (55.1)	0.91	0.75–1.09	0.292
Peru	102 (47.2)	114 (52.8)	0.87	0.73–1.04	0.123
Bolivia	63 (41.4)	89 (58.6)	0.96	0.80–1.16	0.691
Ecuador	25 (23.6)	81 (76.4)	1.26	1.07–1.48	0.007
Mexico	33 (37.5)	55 (62.5)	1.03	0.84–1.26	0.791
**Self-reported anxiety**					
No	349 (42.2)	478 (57.8)	Ref.		
Yes	100 (34.4)	191 (65.6)	1.14	1.03–1.26	0.014
**Self-reported depression**					
No	381 (41.9)	529 (58.1)	Ref.		
Yes	68 (32.7)	140 (67.3)	1.16	1.04–1.29	0.009
**Self-reported stress**					
No	360 (42.7)	484 (57.3)	Ref.		
Yes	89 (32.5)	185 (67.5)	1.18	1.06–1.30	0.001
**Self-reported suicidal ideation**					
No	438 (40.6)	642 (59.4)	Ref.		
Yes	11 (29.0)	27 (71.1)	1.20	0.97–1.47	0.094

High anticipatory perceived labor vulnerability was defined as reporting “quite a lot” or “a lot” in the overall perceived labor impact item. Crude prevalence ratios (cPRs), 95% confidence intervals (95% CIs), and p-values were estimated using generalized linear models with Poisson family, log link, and robust variance. Age and work experience were analyzed as continuous variables; medians and interquartile ranges are shown for descriptive purposes. Estimates represent associations within the analytical sample and should not be interpreted as causal effects

In the multivariable analysis (Table 4), male sex was associated with a lower prevalence of high anticipatory perceived labor vulnerability (aPR: 0.77; 95% CI: 0.69–0.85; *p* < 0.001). Age was not associated with the outcome (aPR: 1.00; 95% CI: 0.99–1.00; *p* = 0.234). Country of residence was not associated with high anticipatory perceived labor vulnerability in the main model. Ecuador showed a higher point estimate compared with the grouped “Other” category, but the confidence interval included the null value (aPR: 1.17; 95% CI: 0.99–1.38; *p* = 0.059).

**Table 4:** Main sociodemographic multivariable model of high anticipatory perceived labor vulnerability related to the Russia–Ukraine war in the analytical sample

Variable	aPR	95% CI	*p*-value
Male	0.77	0.69–0.85	< 0.001
Age, years	1.00	0.99–1.00	0.234
**Country**			
Other	Ref.		
Colombia	1.01	0.86–1.19	0.892
Paraguay	0.90	0.76–1.08	0.271
Peru	0.88	0.74–1.05	0.152
Bolivia	0.95	0.79–1.14	0.586
Ecuador	1.17	0.99–1.38	0.059
Mexico	1.00	0.81–1.22	0.980

Adjusted prevalence ratios (aPRs), 95% confidence intervals (95% CIs), and p-values were estimated using generalized linear models with Poisson family, log link, and robust variance. The model included sex, age, and country of residence. Analytical sample: *n* = 1,118. Estimates should be interpreted as adjusted associations within the analytical sample and not as causal effects or population-level estimates

Sensitivity analyses supported the consistency of the sex association (Supplementary Table 1). When self-reported stress was added to the sociodemographic model, male sex remained associated with a lower prevalence of high anticipatory perceived labor vulnerability (aPR: 0.78; 95% CI: 0.70–0.86; *p* < 0.001), while stress was positively associated with the outcome (aPR: 1.11; 95% CI: 1.00–1.23; *p* = 0.045). In the occupationally adjusted model, male sex remained associated with lower prevalence (aPR: 0.76; 95% CI: 0.68–0.84; *p* < 0.001), and administrative role was also associated with lower prevalence (aPR: 0.84; 95% CI: 0.77–0.93; *p* = 0.001). In the full exploratory model, male sex (aPR: 0.77; 95% CI: 0.69–0.86; *p* < 0.001) and administrative role (aPR: 0.84; 95% CI: 0.76–0.92; *p* < 0.001) remained associated with the outcome. The ordinal logistic sensitivity analysis using the original five-category outcome showed a consistent inverse association for male sex (Supplementary Table 2), although this analysis was interpreted as exploratory because the proportional odds assumption was not formally tested. Variance inflation factors did not suggest severe multicollinearity; the mean VIF was 1.91, with the highest values observed for age and work experience.

## Discussion

The present study found that a substantial proportion of participants in this non-probabilistic Latin American sample reported high anticipatory perceived labor vulnerability related to the Russia–Ukraine war during the early weeks of the conflict. Nearly six out of ten participants classified the possible labor-related impact as “quite a lot” or “a lot”, and most anticipated adverse salary-related consequences. These findings should be interpreted as self-reported, anticipatory perceptions rather than as evidence of actual employment loss, wage reduction, or direct war-related exposure. Nevertheless, they suggest that geographically distant armed conflicts may be accompanied by heightened perceived occupational and economic vulnerability among surveyed individuals in regions already exposed to structural labor fragility and recent socioeconomic disruption.

The Russia–Ukraine war was widely described as a global economic shock because of its potential implications for energy markets, grain exports, fertilizers, trade flows, commodity prices, inflationary pressure, and supply chains. Although the direct humanitarian and productive destruction was concentrated in Ukraine and surrounding regions, macroeconomic modelling and international analyses have shown that the socioeconomic consequences of the war could extend across sectors and regions, including vulnerable economies outside Europe [13–15]. In the present study, these pathways should be understood as contextual conditions that may have shaped public risk appraisal and perceived economic threat, rather than as mechanisms directly measured among respondents. This is particularly relevant for Latin America, where the conflict occurred shortly after the acute phase of the COVID-19 pandemic, when labor markets were still recovering from employment losses, reduced household income, increased informality, and weakened social protection. In this context, the high frequency of anticipatory perceived labor vulnerability observed in this study may reflect workers’ appraisal of a new external shock superimposed on pre-existing economic uncertainty, without implying that objective labor-market deterioration occurred among participants.

The timing of data collection is also important for interpretation. The survey was conducted during the early weeks of the invasion, when media coverage, uncertainty, and public attention to the conflict were particularly high. Perceptions reported during this period may therefore reflect acute crisis salience, information exposure, social amplification, and emotional responses to uncertainty, in addition to or instead of stable labor-related expectations. Without longitudinal follow-up, it is not possible to determine whether these perceptions persisted over time or translated into subsequent job insecurity, wage reduction, employment loss, or other objective occupational outcomes.

The main finding was that male participants had a consistently lower prevalence of high anticipatory perceived labor vulnerability compared with female participants. This association remained consistent in the main sociodemographic model and in sensitivity analyses that additionally adjusted for stress, occupational characteristics, and self-reported mental health variables. However, this finding should not be interpreted as evidence that female participants experienced greater objective labor-market deterioration related to the war. Rather, it indicates a difference in self-reported labor-related risk appraisal within this analytical sample.

This result is consistent with broader evidence indicating that risk perception, economic insecurity, employment vulnerability, and psychosocial responses to crises often differ by sex and gender. Previous literature has reported gender differences in risk preferences and economic uncertainty, with women often showing greater sensitivity to economic risk and insecurity [16]. Studies on job insecurity and psychosocial risks have also shown that women and workers in more precarious positions may report greater perceived vulnerability and work-related stress [17, 18]. In addition, the COVID-19 pandemic disproportionately affected women through sectoral job losses, unpaid care responsibilities, and intensified household and economic pressures, particularly in lower-income settings [19]. Therefore, the sex difference observed in this study may plausibly reflect gendered patterns in perceived economic threat, household responsibility, labor-market vulnerability, reporting behavior, or risk appraisal. However, because the present study did not measure caregiving responsibilities, household income, contract type, informality, sector-specific exposure, media exposure, prior economic vulnerability, or actual job loss, this interpretation must remain cautious and hypothesis-generating.

In crude analyses, self-reported anxiety, depression, and stress were associated with a higher prevalence of high anticipatory perceived labor vulnerability. When stress was added to the sociodemographic model, it remained positively associated with the outcome, although this model was considered a sensitivity analysis rather than the primary model. Conceptually, these variables may not function as conventional confounders. Instead, they may represent related psychosocial constructs, early responses to perceived economic threat, or part of the same broader domain of uncertainty and crisis appraisal. Because anxiety, depression, stress, and suicidal ideation were measured using single binary self-reported items rather than validated clinical scales, these associations should be interpreted as exploratory and susceptible to misclassification. In addition, temporality cannot be established: individuals with greater pre-existing distress may have been more likely to perceive labor-related threats, while perceived labor vulnerability may also have contributed to psychological distress.

Prior studies have shown that perceived employability, economic insecurity, and fear of economic crisis are associated with psychological distress and job dissatisfaction [20, 21]. Similarly, research during COVID-19 found that perceived negative work-related impact was linked to poorer mental well-being and self-rated health among employees [21]. In this sense, the observed associations between mental health indicators and anticipatory perceived labor vulnerability reinforce the interpretation of the outcome as a psychosocial and anticipatory perception rather than an objective occupational consequence.

Country-level differences were not consistent in the main adjusted analysis. Although some country-level differences were observed in crude and sensitivity analyses, country of residence was not associated with high anticipatory perceived labor vulnerability in the main model. Ecuador showed a borderline higher prevalence compared with the grouped “Other” category, but this finding should be interpreted with caution because country-level comparisons were based on non-probabilistic samples and may reflect differences in sample composition rather than true population-level differences. Sensitivity analyses using the original 13-country variable suggested that the way countries were grouped could influence some estimates, particularly for countries with small sample sizes. Therefore, the country findings should not be interpreted as comparative national estimates. Instead, they highlight the need for future studies using representative sampling frames, country-specific labor-market indicators, and measures of media exposure, inflation expectations, and perceived economic insecurity.

Age was not associated with high anticipatory perceived labor vulnerability in the main model. Although younger participants showed slightly higher perceived impact in some descriptive analyses, this pattern was attenuated after adjustment. This is important because young workers may experience greater labor-market vulnerability in many settings, especially in contexts of informality, temporary employment, and limited work experience, but the present data do not provide strong evidence for that association. Any discussion of age should therefore be framed as contextual rather than as a central empirical finding.

Occupational characteristics provided additional exploratory information. Administrative workers had a lower prevalence of high anticipatory perceived labor vulnerability in crude analysis and in occupationally adjusted sensitivity models. This may suggest that workers in operator or field roles perceived greater vulnerability to external shocks, possibly because these roles may be less adaptable to remote work, more exposed to demand fluctuations, or more dependent on in-person economic activity. However, this interpretation is limited by the absence of detailed information on contract type, income, sector-specific exposure, company size, informality, or social protection. In addition, occupational variables may partly capture structural labor conditions or perceived socioeconomic vulnerability rather than confounders of the sociodemographic associations. Similarly, type of work and health occupation were not clearly associated with the outcome, indicating that the perceived labor vulnerability related to the conflict was not restricted to a single occupational category in this sample.

Sensitivity analyses using the original ordinal outcome supported the direction of the sex association, suggesting that this finding was not solely an artifact of dichotomization. Nevertheless, dichotomizing an ordinal variable reduces information and may obscure gradients across categories; future studies should ideally use validated multi-item scales and ordinal or latent-variable approaches. Because the proportional odds assumption was not formally tested, the ordinal logistic model should be interpreted only as a sensitivity analysis and not as confirmatory evidence.

This study has several limitations. First, the study used a non-random snowball sampling strategy; therefore, the sample is not representative of workers in Latin America or of workers within each participating country. Selection bias is likely, and the total number of individuals who received the survey invitation, response rate, and reasons for non-participation could not be determined. The sample may have disproportionately included younger, digitally connected, urban, educated, or socially engaged individuals, limiting external validity. Therefore, the reported percentages should be interpreted as sample-specific descriptive estimates rather than population-level prevalence estimates. Second, the study was cross-sectional and cannot establish causality, temporality, or changes in employment, income, or working conditions. The survey was conducted during the early weeks of the invasion, when media coverage and public uncertainty were particularly high; therefore, responses may have reflected acute crisis salience, information exposure, emotional contagion, or generalized uncertainty rather than stable labor-related expectations. Without longitudinal follow-up, it is not possible to determine whether perceived vulnerability persisted or translated into objective labor outcomes. Third, the primary outcome was subjective, anticipatory, and based on a single non-validated item. It measured perceived possible labor vulnerability rather than actual occupational or economic consequences. This is a substantial methodological limitation because the item may capture heterogeneous constructs, including economic anxiety, inflation concerns, political uncertainty, media-driven threat appraisal, general psychosocial distress, or perceived job insecurity. Consequently, the construct validity, reproducibility, and comparability of the outcome are limited. Future studies should use validated multi-item scales of perceived job insecurity, economic insecurity, risk perception, and psychosocial vulnerability. Fourth, the mental health variables were binary self-reported items from the parent dataset, without available information on exact wording, recall period, severity, or clinical validation, which may have introduced measurement error. These variables should not be interpreted as clinical diagnoses or validated measures of current mental health status. Their associations with perceived labor vulnerability are exploratory and may reflect shared method variance, reverse temporality, or overlapping psychosocial constructs. Fifth, between-country comparisons should be interpreted cautiously because country samples were not representative and smaller countries were grouped into a heterogeneous “Other” category. Sixth, six participants had missing occupation/labor category data, although the primary outcome and main model covariates were complete for all 1,118 participants. Finally, although multicollinearity assessment did not suggest severe collinearity and several sensitivity analyses were performed, interaction effects were not formally assessed, and residual confounding remains likely because relevant variables such as income, contract type, informality, household responsibilities, sector-specific exposure, media exposure, inflation expectations, social protection, household economic dependency, and previous economic vulnerability were not available.

Despite these limitations, this study provides preliminary evidence on how a distant armed conflict may be perceived as a potential labor and economic threat by surveyed workers in a structurally vulnerable regional context. Its main contribution is not the measurement of actual labor-market effects of the Russia–Ukraine war, but the characterization of perceived and anticipatory labor vulnerability in a region exposed to recent pandemic-related disruption and structural employment insecurity. The analysis indicates that high anticipatory perceived labor vulnerability was common within this analytical sample and that sex was the most consistent sociodemographic factor associated with this perception. Future research should use representative samples, validated measures of perceived job insecurity and economic threat, longitudinal designs, measures of information exposure and economic expectations, and objective labor outcomes to better understand how global geopolitical crises shape employment expectations and psychosocial well-being in vulnerable labor markets.

### Supplementary information

The supplementary material includes the Supplementary Methods and Supplementary Tables 1 and 2, which provide additional details on sensitivity analyses, including models adjusted for stress, occupational characteristics, mental health indicators, ordinal outcome specification, multicollinearity assessment, and country-variable specification.

## Supplementary Information

Below is the link to the electronic supplementary material.

Supplementary Material 1

## Data Availability

The dataset supporting the conclusions of this article contains sensitive survey information collected across multiple Latin American countries and is therefore not publicly available due to ethical and confidentiality restrictions. De-identified data may be shared by the corresponding author upon reasonable request, subject to approval by the relevant ethics committee and provided that the requested use is consistent with the original ethical approvals and data protection requirements.
