# Supplementary material for: Sociodemographic factors associated with anticipatory perceived labor vulnerability related to the Russia–Ukraine war among workers in Latin America
Source: Confl Health. 2026 Aug 2;20:86. doi: 10.1186/s13031-026-00823-z (PMC13721457; doi:10.1186/s13031-026-00823-z)
Supplement: Supplementary file 1 [file 13031_2026_823_MOESM1_ESM.docx]

**Supplementary Methods**

Additional analyses were conducted to assess the robustness of the main findings. The primary multivariable model included sex, age, and country of residence. Sensitivity analyses included: (1) a sociodemographic model additionally adjusted for self-reported stress; (2) an occupationally adjusted model including labor category, work experience, type of work, and job role; (3) a full exploratory model additionally including self-reported anxiety, depression, stress, and suicidal ideation; and (4) an ordinal logistic regression model using the original five-category perceived labor impact outcome. Multicollinearity was assessed using variance inflation factors obtained from an equivalent linear regression specification. Because only seven country groups were available, models using country-clustered standard errors were considered exploratory and were not used as the main inferential approach.

**Supplementary Table 1. Sensitivity analyses for high perceived labor impact of the Russia–Ukraine war among workers in Latin America**

| **Variable** | **Main model** | **+ Stress** | **+ Occupational** | **Full exploratory** |
| --- | --- | --- | --- | --- |
| Male | 0.77 (0.69–0.85) | 0.78 (0.70–0.86) | 0.76 (0.68–0.84) | 0.77 (0.69–0.86) |
| Age, years | 1.00 (0.99–1.00) | 1.00 (0.99–1.00) | 1.00 (0.99–1.01) | 1.00 (0.99–1.01) |
| Stress | — | 1.11 (1.00–1.23) | — | 1.11 (0.99–1.24) |
| Health occupation | — | — | 0.97 (0.85–1.10) | 0.96 (0.85–1.09) |
| Work experience, years | — | — | 1.00 (0.99–1.01) | 1.00 (0.99–1.01) |
| Virtual work | — | — | 0.89 (0.71–1.11) | 0.88 (0.70–1.10) |
| Mixed work | — | — | 0.99 (0.87–1.13) | 0.99 (0.87–1.12) |
| Administrative role | — | — | 0.84 (0.77–0.93) | 0.84 (0.76–0.92) |
| Anxiety | — | — | — | 0.99 (0.87–1.13) |
| Depression | — | — | — | 1.06 (0.92–1.22) |
| Suicidal ideation | — | — | — | 1.06 (0.84–1.32) |
| *Values are adjusted prevalence ratios with 95% confidence intervals. All models used generalized linear models with Poisson family, log link, and robust variance. Country coefficients are not shown to preserve compactness. Main and stress models used n=1,118; occupational and full models used n=1,112 because six participants had missing information for occupation.* | | | | |

**Supplementary Table 2. Ordinal logistic regression using the original five-category perceived labor impact outcome**

| **Variable** | **Ordinal main coefficient (SE)** | **p main** | **Ordinal full coefficient (SE)** | **p full** |
| --- | --- | --- | --- | --- |
| Male | -0.676 (0.111) | <0.001 | -0.663 (0.115) | <0.001 |
| Age, years | -0.002 (0.006) | 0.770 | 0.009 (0.013) | 0.509 |
| Administrative role | — | — | -0.269 (0.113) | 0.017 |
| Stress | — | — | 0.218 (0.141) | 0.123 |
| Anxiety | — | — | 0.001 (0.157) | 0.995 |
| Depression | — | — | 0.088 (0.172) | 0.608 |
| Suicidal ideation | — | — | -0.094 (0.326) | 0.773 |
| *The ordinal outcome retained the original five response categories: none, little, fair, quite a lot, and a lot. The ordinal analysis was considered exploratory because the proportional odds assumption was not formally tested.* | | | | |
